# Characterization of the Ocular Surface Microbiome in Keratitis Patients after Repeated Ophthalmic Antibiotic Exposure

**DOI:** 10.1128/spectrum.02162-21

**Published:** 2022-04-04

**Authors:** Yutong Kang, Leihao Tian, Xiaobin Gu, Yiju Chen, Xueli Ma, Shudan Lin, Zhenjun Li, Yongliang Lou, Meiqin Zheng

**Affiliations:** a Wenzhou Key Laboratory of Sanitary Microbiology, Key Laboratory of Laboratory Medicine, Ministry of Education, School of Laboratory Medicine and Life Sciences, Wenzhou Medical University, Wenzhou, Zhejiang, China; b State Key Laboratory for Infectious Disease Prevention and Control, National Institute for Communicable Disease Control and Prevention, Chinese Center for Disease Control and Prevention, Beijing, China; c Optometry and Eye Hospital and School of Ophthalmology, School of Biomedical Engineering, Wenzhou Medical University, Wenzhou, Zhejiang, China; Lerner Research Institute

**Keywords:** keratitis, antibiotic exposure, metagenomic analysis, ocular surface microbiome

## Abstract

In human medicine, antibiotics have been widely used to treat microbial infections. The extensive use of antibiotics is a leading cause of antibiotic resistance. Currently, the influence of the use of antibiotics on the ocular surface microbiome in the course of keratitis treatment remains to be explored in depth. We performed metagenomic analyses in a cohort of 26 healthy controls (HCs), 28 keratitis patients (KPs) who received antibiotics [KP (abx+) group], and 12 KPs who were antibiotic naive [KP (abx−) group]. We identified that the dissimilarities in microbial community structure (Bray-Curtis and Jaccard analyses) between the KP (abx+) group and the HC group were greater than those between the KP (abx−) group and the HC group. Pseudomonas lactis, P. aeruginosa, Pseudomonas sp. *FDAARGOS_380*, Pseudomonas sp. *J380*, Corynebacterium simulans, Streptococcus pyogenes, Finegoldia magna, and Aspergillus oryzae had no statistically significant differences between the KP (abx+) and KP (abx−) groups but did have statistically significant differences between the KP (abx+) and HC groups and between the KP (abx−) and HC groups. Among them, Pseudomonas lactis, P. aeruginosa, Pseudomonas sp. *FDAARGOS_380*, and Pseudomonas sp. *J380* were identified as possible hosts carrying multidrug-resistant genes. The total abundance and number of antibiotic resistance genes (ARGs) were greater in the KP (abx+) group than in the HC and KP (abx−) groups. The functional profile analysis of ocular surface microbiota revealed that pathogenesis-related functional pathways and virulence functions were enriched in KPs. In conclusion, our results show that empirical antibiotic treatment in KPs leads to increases in the antibiotic resistance of ocular surface microbiota.

**IMPORTANCE** Treatment for keratitis is based on appropriate antimicrobial therapy. A direct correlation between antibiotic use and the extent of antibiotic resistance has been reported. Therefore, knowledge of the antibiotic resistance patterns of ocular surface microbial flora in KPs is important for clinical treatment. To the best of our knowledge, this is the first study to use metagenomic approaches to investigate the associations between ophthalmic antibiotic use and the ocular surface microbiome of KPs. Monitoring the microbiota and antibiotic resistome profiles for the ocular surface has huge potential to help ophthalmologists choose the appropriate antibiotics and will thereby improve the efficacy of treatment regimens, which has important implications for reducing the development of antibiotic resistance of the ocular surface to a certain extent.

## INTRODUCTION

Infectious keratitis (IK) is the most frequent cause of corneal blindness in both developing and developed countries. IK is a common yet potentially vision-threatening ophthalmological illness characterized by acute ocular pain, decreased visual acuity, corneal ulcers, and/or infiltrated stroma (1). IK can be caused by a wide variety of microbes, including bacteria, fungi, viruses, parasites, and polymicrobial infections. Studies have shown that polymicrobial infections account for approximately 2 to 15% of all IK cases (2–5). The gold standard for IK treatment is broad-spectrum topical antibiotic therapy. According to the severity of the disease and clinicians’ preference, antibiotic treatment options commonly include monotherapy using fluoroquinolone or dual therapy using a cephalosporin and an aminoglycoside (6).

The spread of antibiotic resistance has been identified as a major global health threat. In recent decades, the discovery of significant antibiotic resistance among ocular pathogens is particularly worrisome (7–10). The easy availability and general acceptance of ophthalmic antibiotics make them susceptible to overuse, which may increase ocular pathogens’ antibiotic resistance (11). The emergence of antibiotic resistance is expected to pose a growing threat to the effective treatment of eye infections (12), which not only may lead to treatment failure but also may make the choice of antibiotics complicated in clinical practice (13–16). Antibiotic resistance should be an important consideration in the treatment of infectious ocular disease, but conventional culture and drug sensitivity testing can take several days to yield results, thus delaying treatment. Therefore, in routine practice, for community-acquired bacterial keratitis physicians often prescribe antibiotics empirically as initial treatment without obtaining smears or cultures (17). Unfortunately, this improper use of antibiotics contributes to the development of antibiotic-resistant bacteria (18).

Past investigations based on culture methods suggest that antibiotic exposure may directly affect the emergence of antibiotic-resistant bacterial strains (19–21). However, conventional culture-based methods are time-consuming, with a low positivity rate; therefore, they cannot provide a comprehensive view of antibiotic resistance genes (ARGs) in complex microbial communities. Limitations of culture methods and amplification-based methods, such as PCR and quantitative PCR, include low throughput, limited availability of primers, biased amplification, false-negative results caused by PCR inhibition, and false-positive results caused by nonspecific amplification (22). High-throughput-sequencing-based metagenomic analysis overcomes these limitations and provides a more global picture of the resistome catalog for the human ocular surface microbiota.

Little has been reported concerning the effects of antibiotic exposure on the occurrence of ARGs in keratitis patients (KPs). Therefore, a metagenomic approach was used in this study to evaluate the effects of antibiotic therapy on the ocular surface microbiota, antibiotic resistome, and function in KPs. This, to the best of our knowledge, represents the beginning of applying metagenomics to the surveillance of the microbial resistome in ophthalmic infections. The application of metagenomic sequencing in infective eye disease can help ophthalmologists choose the appropriate antibiotics for treatment to improve the therapeutic effect.

## RESULTS

### Microbial communities on the ocular surface among the HC group, KP (abx+) group, and KP (abx−) group. (i) Alpha diversity and beta diversity of the microbial communities on the ocular surface.

**FIG 1:**
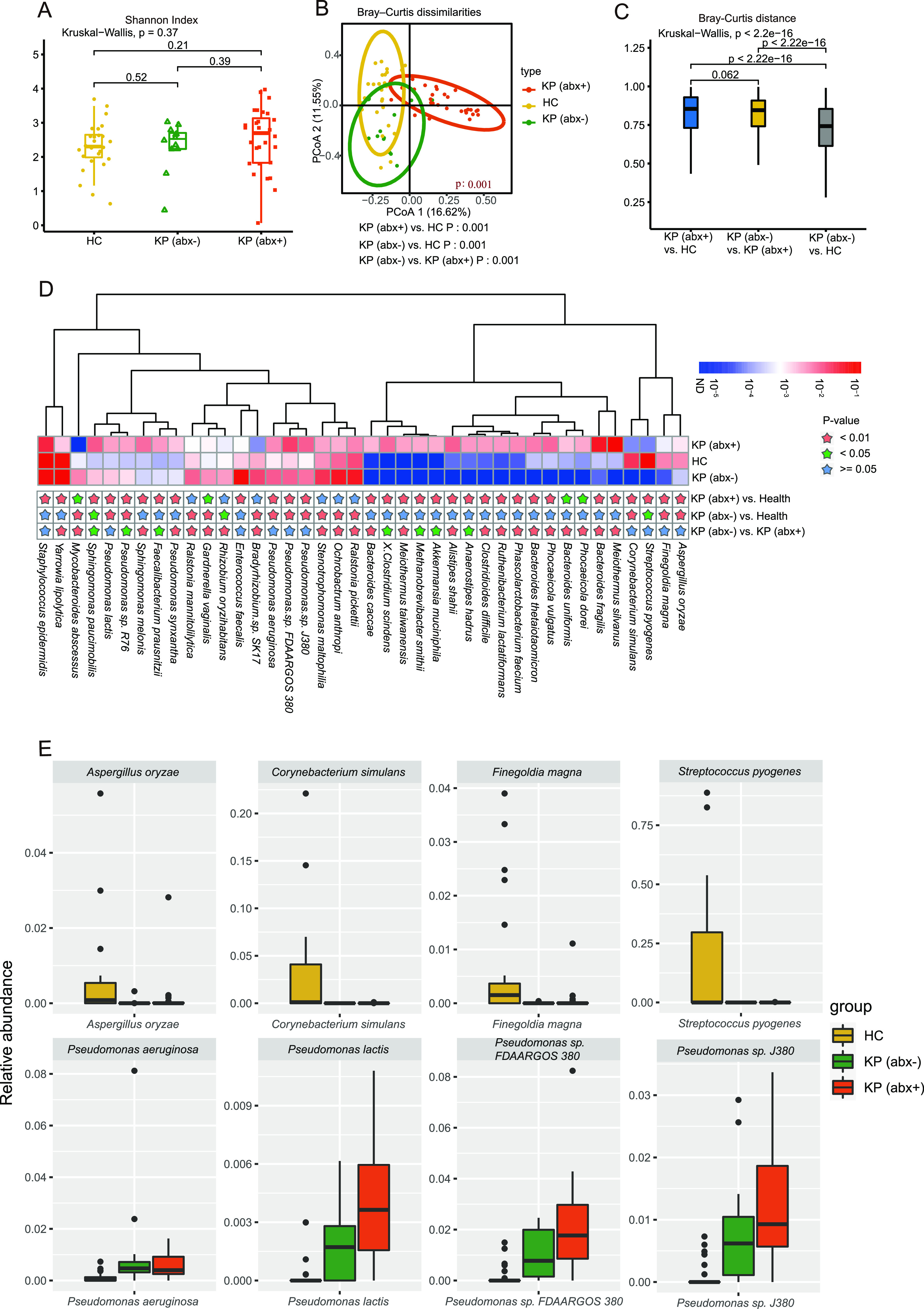
Microbial communities on the ocular surfaces of the HC group, KP (abx+) group, and KP (abx−) group. (A) Shannon-Wiener indexes for the three groups, i.e., HC group, KP (abx+) group, and KP (abx−) group, were compared at the species level. (B) PCoA plot of Bray-Curtis distances for subjects among the three groups. The *P* values from the Kruskal-Wallis test comparing the three groups and the Wilcoxon test comparing two groups are shown. (C) Between-group Bray-Curtis distance comparisons among the HC group, KP (abx+) group, and KP (abx−) group. (D) Relative abundance of the top 40 most different species across the groups. Species with *P* values of <0.01 are labeled with a pink star, those with *P *values of <0.05 with a green star, and those with *P* values of ≥0.05 with a blue star. *P* values are from the Wilcoxon test. (E) Box plots show the distribution of the relative abundance of eight different species among the three groups.

In total, 233,450,474 high-quality nonhuman sequences (average of 3,537,128 sequences per sample) were obtained for further analysis. No statistical difference was observed in the Shannon index (Fig. 1A) or Simpson index (see Fig. S1A in the supplemental material) of ocular surface microbial floras at the species level among the three groups (Kruskal-Wallis test, *P > *0.05). However, there were significant differences in community structure (Bray-Curtis test and permutational multivariate analysis of variance [PERMANOVA] for Bray-Curtis dissimilarity index, *P < *0.05) (Fig. 1B) and composition (Jaccard test and PERMANOVA for Jaccard index, *P < *0.05) (see Fig. S1B) between any two of the three groups at the species level. Beta distances between the KP (abx+) group and HC group communities were significantly greater than distances between the KP (abx−) group and HC group communities for both Bray-Curtis (Fig. 1C) and Jaccard (see Fig. S1C) analyses (Bray-Curtis analysis, Wilcoxon test, *P < *0.05; Jaccard analysis, Wilcoxon test, *P < *0.05). Beta distances between the KP (abx+) group and HC group communities were not dramatically different from distances between the KP (abx+) group and KP (abx−) group communities for both Bray-Curtis and Jaccard analyses (Bray-Curtis analysis, Wilcoxon test, *P > *0.05; Jaccard analysis, Wilcoxon test, *P > *0.05).

### (ii) Identification of differentially abundant microbial signatures.

Figure 1D shows that 40 species were differentially enriched in the HC, KP (abx+), and KP (abx−) groups (Wilcoxon test, *P < *0.05). Pseudomonas lactis, P. aeruginosa, Pseudomonas sp. *FDAARGOS_380*, Pseudomonas sp. *J380*, Corynebacterium simulans, Streptococcus pyogenes, Finegoldia magna, and Aspergillus oryzae had no significant differences between the KP (abx+) and KP (abx−) groups but did have significant differences between the KP (abx+) and HC groups and between the KP (abx−) and HC groups. Compared with the HC group, Pseudomonas lactis, P. aeruginosa, Pseudomonas sp. *FDAARGOS_380*, and Pseudomonas sp. *J380* were significantly increased in the KP (abx+) and KP (abx−) groups, whereas C. simulans, S. pyogenes, F. magna, and A. oryzae were significantly decreased (Fig. 1E). Most notably, the keratitis-enriched species Pseudomonas lactis, P. aeruginosa, Pseudomonas sp. *FDAARGOS_380*, and Pseudomonas sp. *J380* had significant negative correlations with the keratitis-depleted species C. simulans, S. pyogenes, and F. magna (see Fig. S1D).

### (iii) Comparison of traditional culture results with metagenomic data.

Six of 28 cases (21%) in the KP (abx+) group were culture positive, all of which were monomicrobial. Specimens from cases KP (abx+) 2, KP (abx+) 13, KP (abx+) 14, KP (abx+) 17, KP (abx+) 18, and KP (abx+) 24 were culture positive for Acinetobacter baumannii, Fusarium, Streptococcus mitis, P. aeruginosa, Fusarium, and Fusarium, respectively. In those six culture-positive samples, some metagenomic reads were identified as A. baumannii, Fusarium, S. mitis, P. aeruginosa, Fusarium, and Fusarium, respectively, in agreement with the culture results.

Six of 12 cases (50%) in the KP (abx−) group were culture positive, all of which were monomicrobial. Specimens from cases KP (abx−) 1, KP (abx−) 2, KP (abx−) 9, KP (abx−) 10, KP (abx−) 11, and KP (abx−) 12 were culture positive for Aspergillus, Corynebacterium accolens, Staphylococcus epidermidis, Fusarium, Streptococcus oralis, and P. aeruginosa, respectively. With the exception of the KP (abx−) 2 sample, metagenomic sequencing could identify bacteria found by culture in the culture-positive samples. These results suggest that metagenomic sequencing and culture present good concordance.

### ARGs in ocular surface communities among the HC group, KP (abx+) group, and KP (abx−) group. (i) Broad-spectrum profiles of ARG abundance.

**FIG 2:**
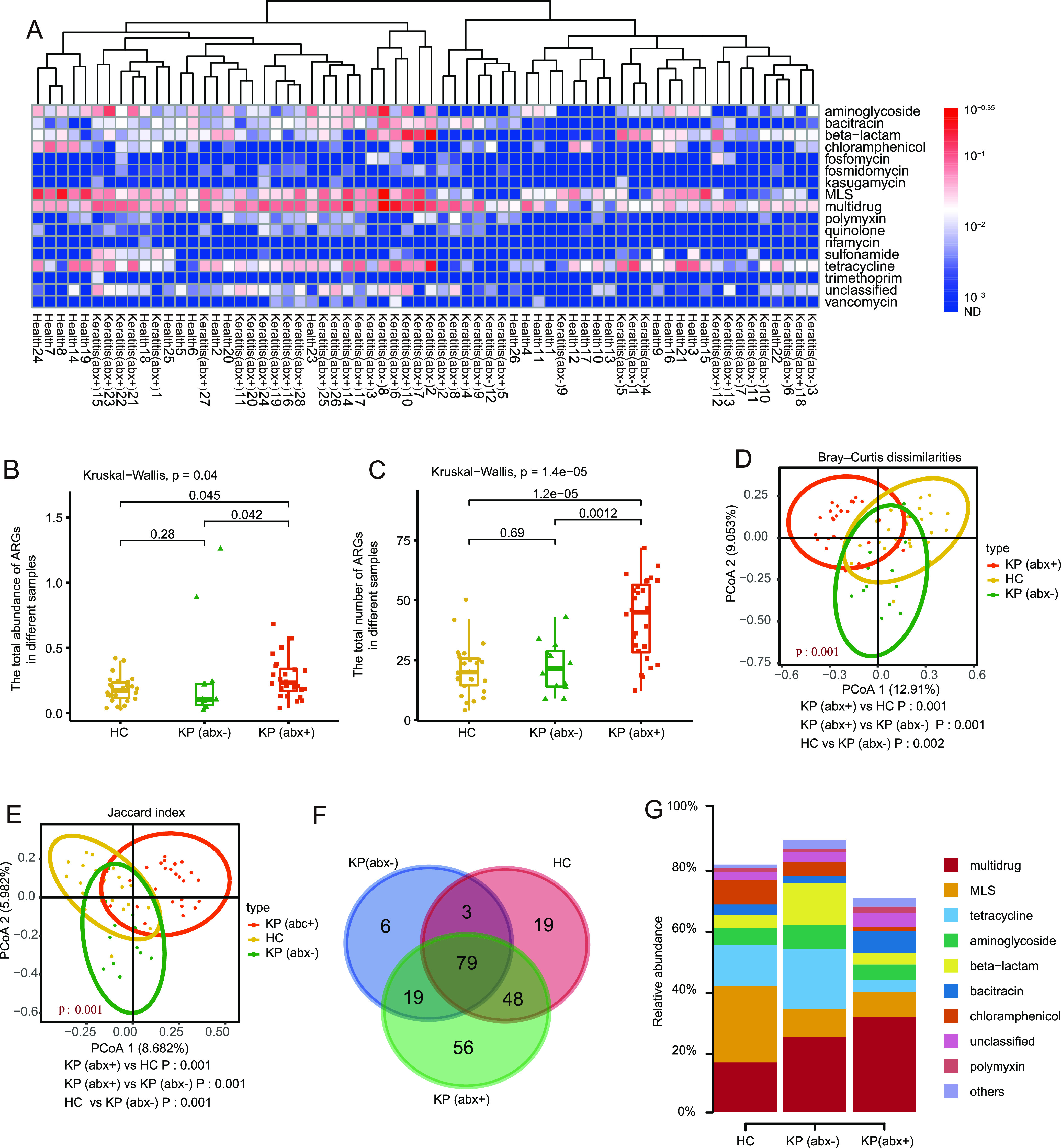
Antibiotic resistome differences among the HC group, KP (abx+) group, and KP (abx−) group. (A) Broad-spectrum quantitative profiles of the ARG types (copy per 16S rRNA) in 66 subjects. (B and C) Box plots of the abundance (B) and number (C) of ARG subtypes in the HC group, KP (abx+) group, and KP (abx−) group. (D) PCoA of Bray-Curtis distances between groups. (E) PCoA of Jaccard distances between groups. (F) Venn diagram showing the numbers of shared and unique ARG subtypes among the HC group, KP (abx+) group, and KP (abx−) group. (G) Relative abundance of shared ARGs. The *P* values from the Kruskal-Wallis test comparing the three groups and the Wilcoxon test comparing two groups are shown.

In the present study, the SARG database, which is composed of 24 ARG types and 1,244 subtypes, was applied to perform the ARG search. Compared with the traditional culture method and PCR, our results provided a more global picture of the resistome catalog for the human ocular surface microbiota. A total of 230 ARG subtypes belonging to 17 ARG types were detected. The total ARG abundances (copy of ARG per copy of 16S rRNA gene) of the 66 subjects from the three groups were 0.040 to 0.684 in the KP (abx+) group, 0.036 to 0.421 in the HC group, and 0.020 to 1.260 in the KP (abx−) group. In general, the resistance genes for multidrug, macrolide-lincosamide-streptogramin (MLS), tetracycline, β-lactam, aminoglycoside, and bacitracin resistance were more abundant than the other ARG types in these samples (Fig. 2A). Figure S2 in the supplemental material shows that 96 ARG subtypes were present in at least 10% of samples, which were considered the representative ARGs.

### (ii) Antibiotic resistome differences among the HC group, KP (abx+) group, and KP (abx−) group.

As shown in Fig. 2B and C, the total ARG abundances and numbers in the KP (abx+) group were significantly greater than those in the HC group (Wilcoxon test, *P < *0.05) and the KP (abx−) group (Wilcoxon test, *P < *0.05). Notably, no difference in the total ARG abundances and numbers between the HC group and the KP (abx−) group was detected (Wilcoxon test, *P > *0.05). Principal-coordinate analysis (PCoA) (Bray-Curtis test and PERMANOVA for Bray-Curtis dissimilarity index, *P < *0.05 [Fig. 2D]; Jaccard test and PERMANOVA for Jaccard index, *P < *0.05 [Fig. 2E]) revealed that the samples from different groups clustered more closely and had a significant difference in ARG compositions.

### (iii) Shared ARGs among the HC group, KP (abx+) group, and KP (abx−) group.

Overall, 149, 107, and 202 ARGs were identified in the HC group, KP (abx−) group, and KP (abx+), respectively (Fig. 2F). There were 19, 6, and 56 unique ARGs in the HC group, KP (abx−) group, and KP (abx+) group, respectively. Venn diagram analysis indicated that 79 ARGs were common to all subjects; therefore, these ARGs represented a “core resistome.” The core ocular surface resistome was evidently composed of genes from the multidrug, MLS, tetracycline, aminoglycoside, and β-lactam resistance classes. The shared ARGs accounted for 80.97 ± 10.84%, 88.93 ± 7.67%, and 70.02 ± 17.19% of the total abundances of ARGs detected in the HC group, KP (abx−) group, and KP (abx+) group, respectively (Fig. 2G).

### (iv) Correlation network of cooccurring ARG subtypes and microbial taxa.

**FIG 3:**
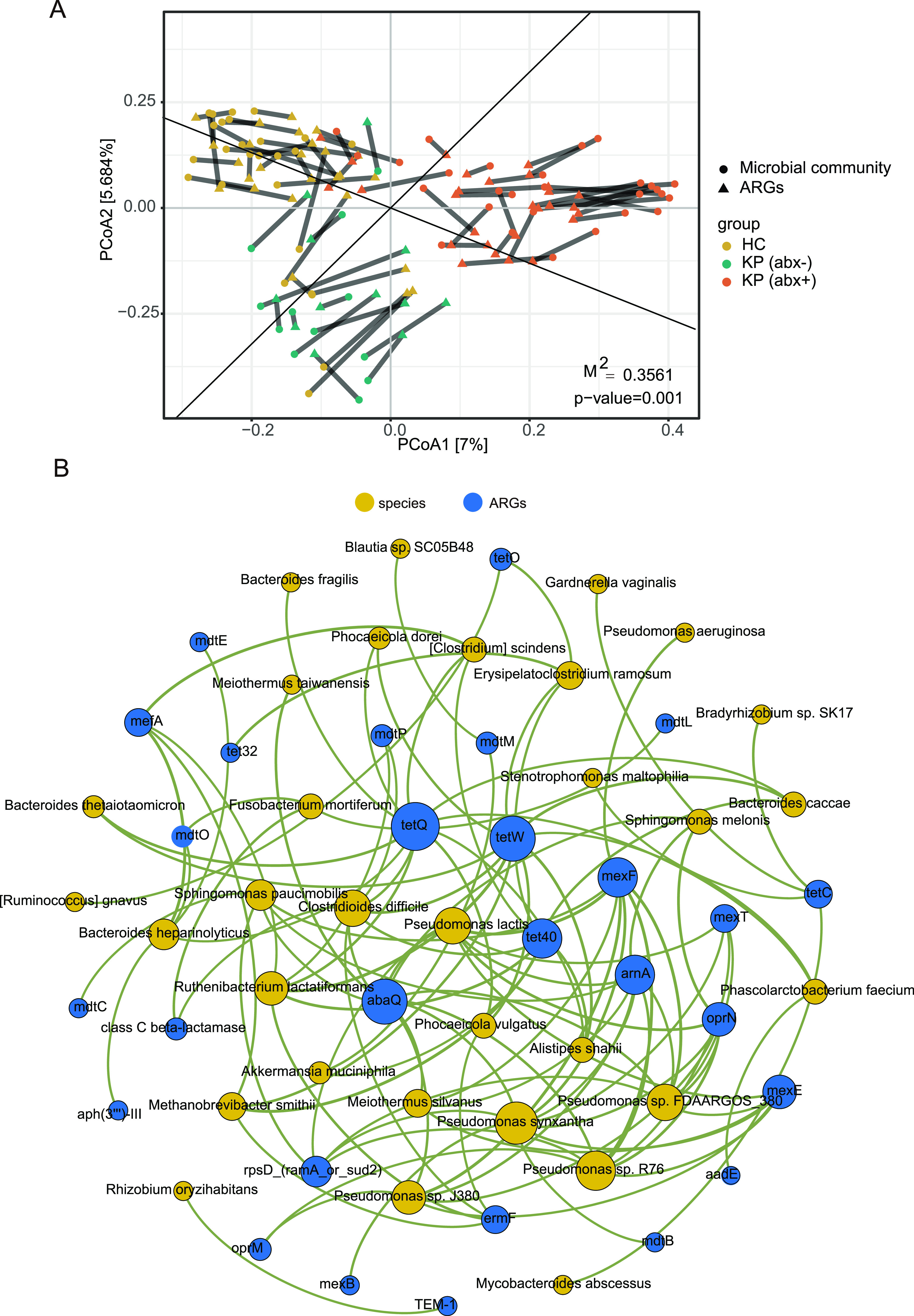
(A) Procrustes analysis of the correlations between antibiotic resistome and microbiota structures based on a Bray-Curtis dissimilarity matrix. (B) Network analysis showing the cooccurrence pattern between ARG subtypes and microbial taxa based on Spearman’s correlation analysis. A connection represents a strong and significant correlation (FDR adjusted, *P < *0.01, Spearman’s *r *> 0.6). The size of each node is proportional to the number of connections. The species nodes are colored yellow, and the ARG nodes are colored blue.

Procrustes analysis provided the overall correlation between the antibiotic resistome and the microbial communities. As shown in Fig. 3A, on the basis of the PCoA of both the ARG subtype abundances and the species abundances, the result of the Procrustes analysis showed a significant correlation between the antibiotic resistome and the microbial communities in different samples (Procrustes test, 999 permutations, *m*^2^ = 0.4149, *P < *0.05), which indicated that there was a significant consistency between microbial communities and ARGs. In total, 28 ARGs and 32 microbial taxa were screened based on the strong (ρ > 0.6) and significant (false-discovery rate [FDR] adjusted, *P < *0.01) Spearman’s rank correlations (Fig. 3B; also see Fig. S3) to perform network analyses. There were 60 nodes and 109 edges in the network. The detailed cooccurrence between microbial taxa and ARG subtypes is presented in Table S1 in the supplemental material.

The modularity index of 0.575 (>0.4) indicated that the positive network possessed a modular structure (23). Based on the modularity class, the full network could be parsed into seven major modules, ranging in size from 2 to 21 nodes. Notably, all Pseudomonas species in the network and the ARGs they carry are in the same module. Even following antibiotic treatment, the KP (abx+) group still had greater abundance of Pseudomonas lactis, P. aeruginosa, Pseudomonas sp. *FDAARGOS_380*, and Pseudomonas sp. *J380*, compared with the HC group (Fig. 1D). Highly significant positive correlations were observed between pairs of the keratitis-enriched species Pseudomonas lactis, P. aeruginosa, Pseudomonas sp. *FDAARGOS_380*, and Pseudomonas sp. *J380* (see Fig. S1D). In the correlation network of cooccurring ARG subtypes and microbial taxa, these four Pseudomonas species and the ARGs they carry are also in the same module. One possible explanation for related cooccurring ARGs and microbial taxa in each module is that they might often be harbored simultaneously.

According to the hypotheses formulated by Li et al. (22), if the ARGs and coexisting microbial taxa possessed significantly similar abundance tendencies among the different samples, then the microbial taxa could potentially be ARG hosts. As presented in Table S1 in the supplemental material, 32 species were speculated to be the possible hosts of 28 ARGs, based on the cooccurrence results. Regarding genera, Pseudomonas mainly carried multidrug (*mdtB*, *mexB*, *mexE*, *mexF*, *mexT*, *oprN*, and *oprM*), polymyxin (*arnA*), quinolone (*abaQ*), and unclassified (*rpsD*) resistance genes. It is noteworthy that Pseudomonas sp. *FDAARGOS_380*, Pseudomonas sp. *J380*, P. aeruginosa, Pseudomonas lactis, Pseudomonas sp. *R76*, and Pseudomonas synxantha all potentially carried multidrug resistance subtypes (*mexF*). Some species (Clostridioides difficile, Pseudomonas lactis, Pseudomonas sp. *FDAARGOS_380*, Pseudomonas sp. *J380*, Pseudomonas sp. *R76*, P. synxantha, and Ruthenibacterium lactatiformans) carried six or more ARGs. Other species were associated with five or fewer ARG subtypes.

### Functional composition differences among the HC group, KP (abx+) group, and KP (abx−) group.

**FIG 4:**
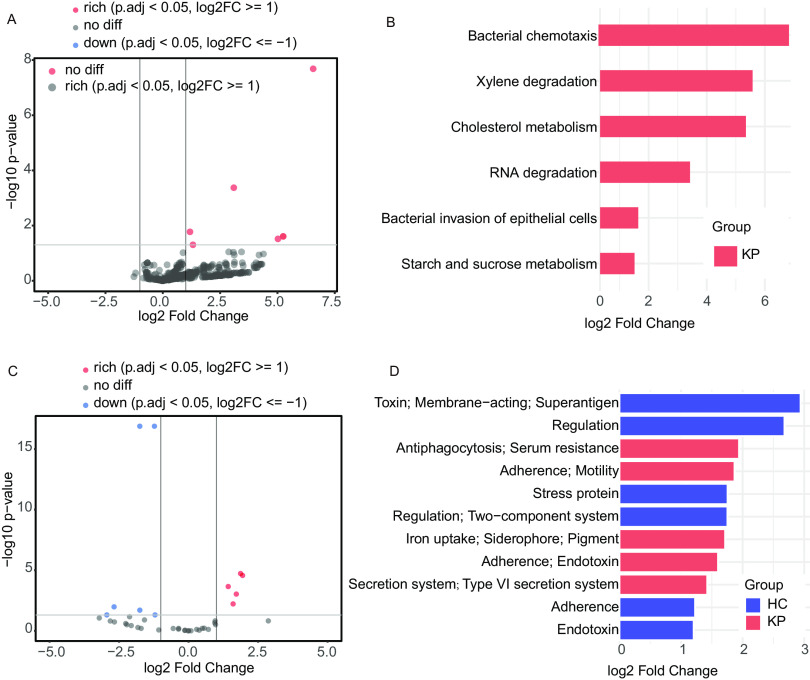
(A and B) Volcano plot (A) and corresponding bar plot (B) of KEGG pathway function differential analysis comparing samples from HCs (*n* = 26) versus KPs (*n* = 40). (C and D) Volcano plot (C) and corresponding bar plot (D) of predicted virulence function differential analysis comparing samples from HCs (*n* = 26) versus KPs (*n* = 40). KEGG pathway functions and predicted virulence functions with log_2_ fold changes of >1 and adjusted *P* values of <0.05 were considered significant (solid gray lines). The statistical test was the Wald test in DeSeq2. The red color indicates an increase in the KP group versus the HC group. The blue-purple color indicates a decrease in the KP group versus the HC group. LDA, linear discriminant analysis.

The large quantity of metagenomic shotgun sequencing data obtained in the present study allowed us to determine the changes in the functional pathways and virulence functions encoded in the metagenome in keratitis and healthy states. In the comparison between KPs and HCs, six pathways were significantly enriched in KPs, including bacterial chemotaxis, xylene degradation, cholesterol metabolism, RNA degradation, bacterial invasion of epithelial cells, and starch and sucrose metabolism (Fig. 4A and B). To further investigate the potential virulence mechanisms involved, the virulence functions of ocular surface microbiota were predicted according to the VFDB classification. change the text crossed out to keratitis patients versus healthy controls comparison identified 11 differentially abundant predicted virulence function classifications, among which 5 virulence function classifications were increased in the keratitis patients, such as [antiphagocytosis; serum resistance], [adherence; motility], [iron uptake; siderophore; pigment], [adherence; endotoxin], and [secretion system; type VI secretion system] (Fig. 4C and D).

## DISCUSSION

IK is a vision-threatening emergency requiring urgent ophthalmic attention. Management commonly involves obtaining microbiology specimens using corneal scraping to obtain the results of microbial cultures and drug sensitivity testing. However, in the majority of cases, empirical antibiotics are prescribed by the ophthalmologist in the initial treatment to avoid treatment delays. However, this inappropriate antibiotic utilization might result in an increase in ocular surface microbiota antibiotic resistance. Notably, the antibiotics used act on not only major pathogens but also all flora on the ocular surface, which has an influence on both the composition and antibiotic resistance of the whole ocular surface microbiome. Although several studies have reported the spectra and trends of isolated microorganisms and the antibiotic sensitivity patterns of IK (24–27), the effect of antibiotic use on the resistance of the whole ocular surface microbiome in KPs has received little attention. To the best of our knowledge, this is the first study revealing the impact of antibiotic use on the ocular surface resistome of KPs based on metagenomic sequencing. Paying attention to the ocular surface microbiota and antibiotic resistance is important in guiding the selection of antibiotics for antimicrobial therapy and ultimately improving patient outcomes, which is also of great value in reducing the generation of antibiotic resistance in the ocular surface microbiome in the future.

Our results showed that there was a significant difference in microbial community structure between the KP (abx+) group and the HC group and between the KP (abx+) group and the KP (abx−) group. In addition, the dissimilarities (Bray-Curtis and Jaccard analyses) between the KP (abx+) group and the HC group were greater than the dissimilarities between the KP (abx−) group and the HC group. These results suggested that repeated antibiotic exposure can trigger conspicuous variations in microbial communities, which can further result in the decreased inhibition of pathogenic species and may have important clinical consequences (11). This stresses the necessity for comprehensive knowledge regarding the ocular surface microbial community and antibiotic resistome for proper and rational use of antibiotics in clinical ophthalmological practice.

Even following antibiotic treatment, the KP (abx+) group still had greater abundance of Pseudomonas lactis, P. aeruginosa, Pseudomonas sp. *FDAARGOS_380*, and Pseudomonas sp. *J380*, compared with the HC group. Follow-up analyses revealed that these Pseudomonas species were potential hosts carrying the multidrug resistance genes, which could be an important reason why these species could not be killed by antibiotics. We speculated that biofilm formation could also play a role in antibiotic resistance. It has been reported that 80% of bacterial infections are caused by bacteria living in biofilms (28). The inability of antibiotics to penetrate the Pseudomonas biofilm reduces the ability of antibiotics to kill biofilm bacteria (29, 30). P. aeruginosa is an important etiology associated with IK (31) and is particularly notorious for being able to form biofilms in many environments and causing a wide variety of chronic infections (32). A retrospective study with case series reported that patients with multidrug-resistant P. aeruginosa ocular infections were more likely to experience corneal perforation (33). Despite its low abundance, P. aeruginosa is a ubiquitous pathogen, with 90% positivity rates among our KPs.

Our data suggest a worrying scenario in which Pseudomonas species on the ocular surface of KPs exhibit resistance to multiple antibiotics. In agreement with many past studies, our findings indicate that P. aeruginosa carries the *mexF* resistance gene, and P. synxantha carries the *mdtB*, *mexB*, *mexE*, *mexT*, *oprN*, and *rpsD* resistance genes. Furthermore, to the best of our knowledge, our study is the first to find that Pseudomonas lactis potentially carries the *mexE*, *mexF*, *mexT*, *oprN*, *arnA*, *abaQ*, and *rpsD* resistance genes, Pseudomonas sp. *FDAARGOS_380* potentially carries the *mexE*, *mexF*, *mexT*, *oprN*, *arnA*, *abaQ*, and *rpsD* resistance genes, Pseudomonas sp. *J380* potentially carries the *mexE*, *mexF*, *oprM*, *oprN*, *arnA*, and *abaQ* resistance genes, Pseudomonas sp. *R76* potentially carries the *mexE*, *mexF*, *mexT*, *oprM*, *oprN*, *arnA*, *abaQ*, and *rpsD* resistance genes, and P. synxantha potentially carries the *mexF*, *arnA*, and *abaQ* resistance genes. Therefore, our study adds to the existing body of knowledge on the ARGs of Pseudomonas species.

A key observation from our work is that the number and abundance of total ARGs in the KP (abx+) group were dramatically higher than those in the KP (abx−) group and the HC group but there was no statistical difference in the number and abundance of total ARGs between the KP (abx−) group and the HC group. In prior literature, it has been reported that, although several factors play a promoting role in antibiotic resistance development, inappropriate antibiotic use is the main cause (34, 35). After empirical antibiotic therapy, increasing the number and abundance of ARGs in the ocular surface microbiome of KPs may place patients at risk of developing chronic keratitis that is more difficult to treat.

Innate immune defenses enable the intact corneal epithelium to strongly resist infection despite its continuous exposure to a range of microorganisms, allowing the corneal epithelium to coordinate harmonious symbiosis between the host and environmental microbes (36). At the function level, we found that the ocular surface microbiome of KPs is enriched with genes related to the bacterial invasion of epithelial cells, suggesting that alterations in ocular surface microorganisms could play a potential role in the onset of keratitis or disease progression. In addition, the virulence function associated with adherence is significantly enriched in KPs, and adherence to the surface of epithelial cells is a prerequisite for bacteria to establish infections *in vivo* (36). The enrichment of genes related to bacterial chemotaxis in KPs may play an important role in the initial stage of pathogen infection (37). Regarding our future work, a more detailed transcriptomic and metabolomic study of these phenomena is planned.

### Conclusions.

This study’s findings collectively revealed the effects of empirical antibiotic treatment on the ocular surface microbiome of KPs, which emphasizes the need for accurate and rational use of antibiotics in ophthalmology practices. The composition and antibiotic resistance of the ocular surface microbial community in KPs should be highlighted as an essential consideration in current treatment options.

## MATERIALS AND METHODS

### Study participants.

A total of 66 subjects were enrolled in this study by the Affiliated Eye Hospital of Wenzhou Medical University (Wenzhou, China), including 40 KPs and 26 HCs. All subjects were assessed with required general and ophthalmic examinations. The inclusion criteria for KP selection were as follows: corneal epithelial ulceration (>1 mm in diameter), corneal epithelial defect with infiltration, presence of signs of acute inflammation, and no fungal elements detected by confocal microscopy. All patients had experienced the onset of keratitis within 6 months and had no history of antibiotic or probiotic use, systemic or ocular diseases, or contact lens wear. Among all 40 patients, 28 patients had undergone more than 7 days of antibiotic therapy prior to sample collection, including (i) patients who self-medicated with antibiotics, (ii) patients who had a history of antibiotic treatment in other hospitals, and (iii) patients who accepted empirical topical prescription antibiotic treatment before the culture results were available. The 28 patients who had received antibiotics were designated the KP (abx+) group and were allocated subject numbers from KP (abx+) 1 to KP (abx+) 28. The remaining 12 patients were antibiotic naive; they were designated the KP (abx−) group and were allocated subject numbers from KP (abx−) 1 to KP (abx−) 12. The 26 HCs had no history of antibiotic or probiotic use, systemic or ocular diseases, or contact lens wear within the past 6 months, and they were allocated subject numbers from HC1 to HC26.

This study was approved by the Ethics Committee of Affiliated Eye Hospital of Wenzhou Medical University (approval number KYK [2017] 23). All of the subjects signed an informed consent form upon enrollment. The conduct of the study adhered to the tenets of the Declaration of Helsinki.

### Sample collection, DNA extraction, prevention of contamination, preparation of negative controls, and metagenomic shotgun sequencing.

All sample collection was conducted in an ophthalmic treatment room sterilized by UV irradiation. After the instillation of sterile topical proparacaine, samples were collected from ocular surface mucosal sites, including upper and lower palpebral sites, the caruncle, and the conjunctival fornix, using flocked swabs of the ESwab transport system (Copan Diagnostics Inc., Murrieta, CA, USA). All KPs developed unilateral eye disease. A randomly chosen eye from each HC was sampled as a control. Collected swabs were immediately placed on ice and transferred to the freezer to be frozen at −80°C until processing. Total genomic DNA was extracted according to the DNA extraction kit (product numbers 19092 and 50214; Qiagen, Hilden, Germany). DNA concentration and purity were determined using a Qubit 2.0 fluorometer (Life Technologies, CA, USA). The methods for the prevention of contamination and preparation of negative controls were in accordance with our previously published paper (38). DNA libraries for Illumina sequencing were generated using the NEBNext Ultra DNA library preparation kit (NEB, USA). Libraries were paired-end sequenced on a HiSeq X 10 platform (Novogene Co., Ltd., Beijing, China) with an insert size of 350 bp and a read length of 150 bp.

### Bioinformatic analyses.

The human sequences (according to alignment to hg19), as well as low-quality reads, were discarded by Bowtie2 (39) and SAMtools (40). The high-quality sequences were taxonomically classified using Kraken2 (41). ARG-like reads in all samples were identified and annotated through the online analysis pipeline ARGs-OAP v2.0 (42). The clean nonhuman reads were assembled using MEGAHIT (43). Prokka was used to predict genes (44). Gene abundance was computed using Salmon (45). Deredundancy analysis of predicted genes was then performed using CD-HIT (46). The nonredundant genes were annotated using DIAMOND software (47) against the KEGG database (http://www.genome.jp/kegg) for functional annotation of the metagenomes. Finally, virulence genes were annotated based on the VFDB databases (48).

### Statistical analysis.

Statistical analysis was performed using R software (version 4.1.0). The alpha and beta diversity indices were calculated using the vegan package in R (49). Statistical comparisons between two groups were performed using the Wilcoxon test. Differences among the three groups were statistically examined using the Kruskal-Wallis test. *P* values of <0.05 were considered to indicate statistically significant differences. PCoA was performed to assess the microbial community structures among three groups based on Bray-Curtis dissimilarities and the Jaccard index. PERMANOVA was performed on beta diversity results to determine statistical differences among the groups. All Spearman correlations among the microbial communities and ARGs were calculated in R with the Hmisc package (50). Network visualization was realized using Gephi software (51). The Spearman correlation value (*r*) for each pair of eight different species was investigated with the PerformanceAnalytics package in R (52).

### Data availability.

The metagenomic sequencing data set was deposited in the NCBI Sequence Read Archive (SRA) under BioProject accession number PRJNA774492.
