# Supplementary material for: Characterization of the Ocular Surface Microbiome in Keratitis Patients after Repeated Ophthalmic Antibiotic Exposure
Source: Microbiol Spectr. 2022 Apr 4;10(2):e02162-21. doi: 10.1128/spectrum.02162-21 (PMC9045205; doi:10.1128/spectrum.02162-21)
Supplement: SUPPLEMENTAL FILE 1 — Supplemental material. Download SPECTRUM02162-21_Supp_1_seq14.pdf, PDF file, 6.3 MB [file spectrum02162-21_supp_1_seq14.pdf]

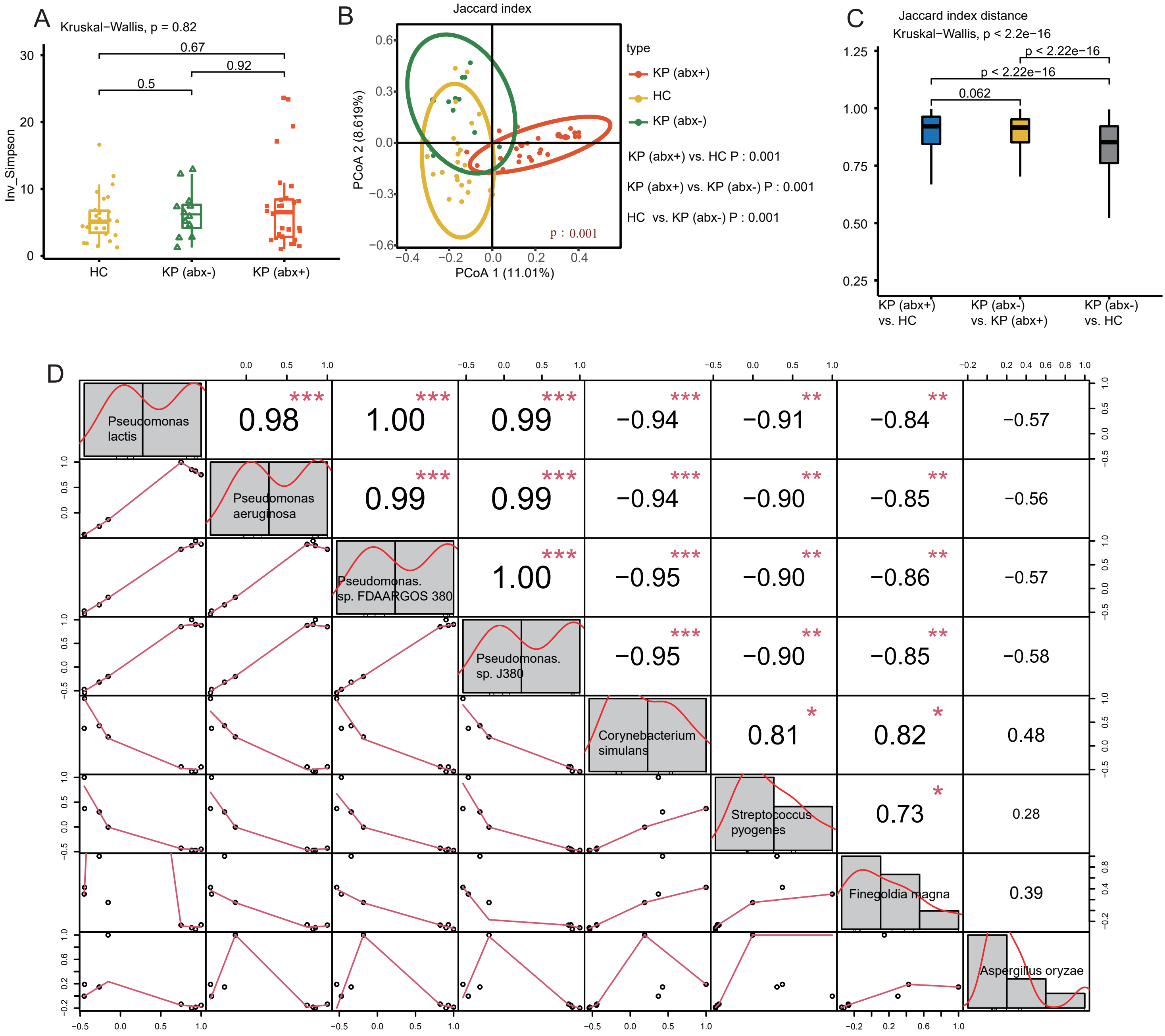

Fig. S1 (A) Simpson indexes of the three groups (HC group, KP (abx+) group, and KP (abx-) group) were compared at the species levels. (B) PCoA plot of Jaccard distances for subjects among the three groups. PERMANOVA, non-parametric permutational multivariate analysis of variance. (C) Between-group Jaccard distance comparisons among HC group, KP (abx+) group, and KP (abx-) group. (D) Pairwise Spearman's correlation analyses among eight different species. Significance level (*P < 0.05; **P < 0.01; ***P < 0.001). The P-values from the Kruskal–Wallis test comparing the significance of three groups and the Wilcox test comparing two groups are shown.

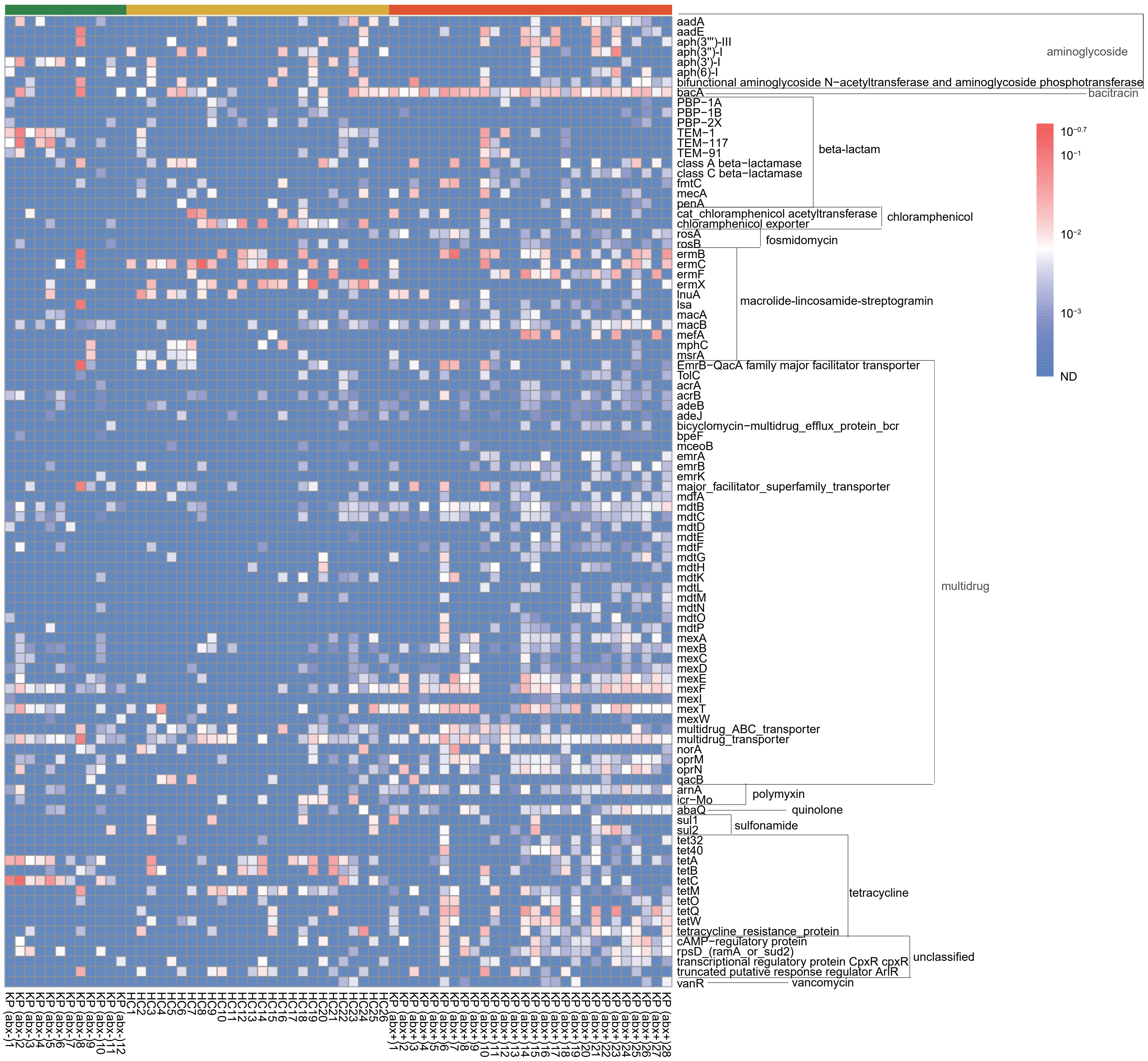

Fig. S2 Abundance of 96 ARG subtypes present in at least 10% of samples (copy/16S rRNA).

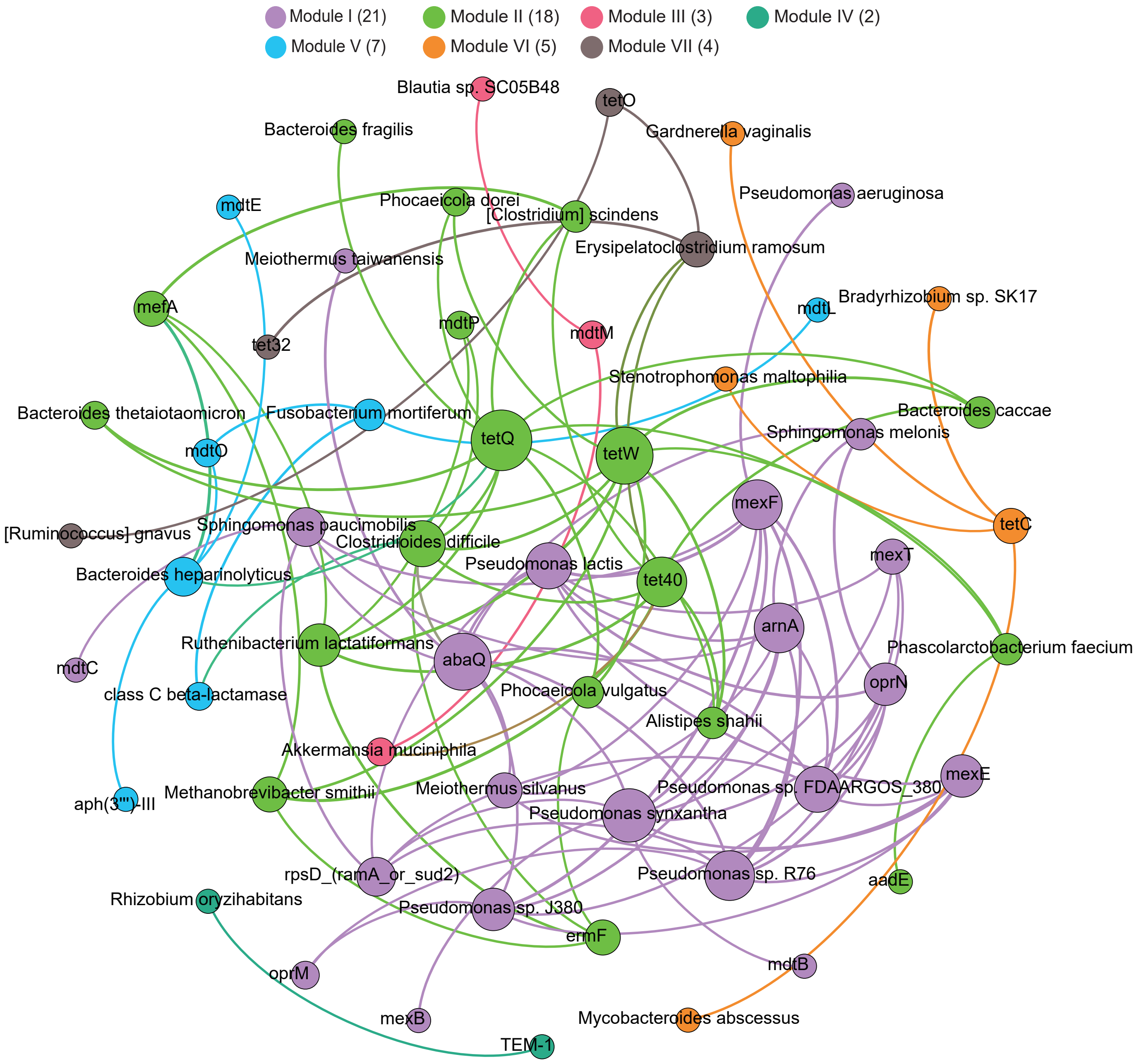

Fig. S3 Network analysis showing the co-occurrence pattern between ARGs subtypes and microbial taxa based on Spearman's correlation analysis. A connection represents strong and significant (FDR adjusted, P-value < 0.01, Spearman's r > 0.6) correlation. The size of each node is proportional to the number of connections. The nodes were colored according to the modularity class.

Table S1 Potential ARG hosts information revealed by co-occurrence between ARG subtypes and microbial taxa.

Species	Genus	ARG subtype	ARG type
Akkermansia muciniphila	Akkermansia	mdtM	multidrug
		tet40	tetracycline
Alistipes shahii	Alistipes	tet40	tetracycline
		tetQ	tetracycline
		tetW	tetracycline
Bacteroides caccae	Bacteroides	tet40	tetracycline
		tetQ	tetracycline
		tetW	tetracycline
Bacteroides fragilis	Bacteroides	tetQ	tetracycline
Bacteroides heparinolyticus	Bacteroides	aph(3''')-III	aminoglycoside
		mefA	MLS
		metE	MLS
		mdtO	multidrug
		tetQ	tetracycline
Bacteroides thetaiotaomicron	Bacteroides	tetQ	tetracycline
		tetW	tetracycline
Blautia sp. SC05B48	Blautia	mdtM	
Bradyrhizobium sp. SK17	Bradyrhizobium	tetC	tetracycline
Clostridioides difficile	Clostridioides	class C beta-lactamase	beta-lactam
		ermF	MLS
		mdtP	multidrug
		abaQ	quinolone
		tet40	tetracycline
		tetQ	tetracycline
		tetW	tetracycline
[Clostridium] scindens	Lachnoclostridium	mefA	MLS
		tet40	tetracycline
		tetQ	tetracycline
Erysipelatoclostridium ramosum	Erysipelatoclostridium	tet32	tetracycline
		tet40	tetracycline
		tetO	tetracycline
		tetW	tetracycline
Fusobacterium mortiferum	Fusobacterium	class C beta-lactamase	beta-lactam
		mdtL	multidrug
		mdtO	multidrug
Gardnerella vaginalis	Gardnerella	tetC	tetracycline
Meiothermus silvanus	Meiothermus	mexE	multidrug
		mexF	multidrug
		arnA	polymyxin
		abaQ	quinolone
Meiothermus taiwanensis	Meiothermus	abaQ	quinolone
Methanobrevibacter smithii	Methanobrevibacter	ermF	MLS
		mefA	MLS
		tet40	tetracycline
		tetW	tetracycline
Mycobacteroides abscessus	Mycobacteroides	tetC	tetracycline
Phascolarctobacterium faecium	Phascolarctobacterium	aadE	aminoglycoside
		tetQ	tetracycline
		tetW	tetracycline
Phocaeicola dorei	Phocaeicola	tetQ	tetracycline
		tetW	tetracycline
Phocaeicola vulgatus	Phocaeicola	ermF	MLS
		tetQ	tetracycline
		tetW	tetracycline
Pseudomonas aeruginosa	Pseudomonas	mexF	multidrug
Pseudomonas lactis	Pseudomonas	mexE	multidrug
		mexF	multidrug
		mexT	multidrug
		oprN	multidrug
		arnA	polymyxin
		abaQ	quinolone
		rpsD	unclassified
Pseudomonas sp. FDAARGOS_380	Pseudomonas	mexE	multidrug
		mexF	multidrug

		mexT	multidrug
		oprN	multidrug
		arnA	polymyxin
		abaQ	quinolone
		rpsD	unclassified
Pseudomonas sp. J380	Pseudomonas	mexE	multidrug
		mexF	multidrug
		oprM	multidrug
		oprN	multidrug
		arnA	polymyxin
		abaQ	quinolone
Pseudomonas sp. R76	Pseudomonas	mexE	multidrug
		mexF	multidrug
		mexT	multidrug
		oprM	multidrug
		oprN	multidrug
		arnA	polymyxin
		abaQ	quinolone
		rpsD	unclassified
Pseudomonas synxantha	Pseudomonas	mdtB	multidrug
		mexB	multidrug
		mexE	multidrug
		mexF	multidrug
		mexT	multidrug
		oprN	multidrug
		arnA	polymyxin
		abaQ	quinolone
		rpsD	unclassified
Rhizobium oryzihabitans	Rhizobium	TEM-1	beta-lactam
[Ruminococcus] gnavus	Mediterraneibacter	tetO	tetracycline
Ruthenibacterium lactatiformans	Ruthenibacterium	ermF	MLS
		mefA	MLS
		mdtP	multidrug
		tet40	tetracycline
		tetQ	tetracycline
		tetW	tetracycline
Sphingomonas melonis	Sphingomonas	oprN	multidrug
		arnA	polymyxin
		abaQ	quinolone
Sphingomonas paucimobilis	Sphingomonas	mdtC	multidrug
		mexF	multidrug
		arnA	polymyxin
		abaQ	quinolone
		rpsD	unclassified
Stenotrophomonas maltophilia	Stenotrophomonas	tetC	tetracycline

MLS: Macrolide-Lincosamide-Streptogramin
